# The synergetic effects of 4-nonylphenol and polyethylene microplastics in *Cyprinus carpio* juveniles using blood biomarkers

**DOI:** 10.1038/s41598-023-38636-2

**Published:** 2023-07-19

**Authors:** Esraa Ammar, Mohamed Hamed, Mahmoud S. Mohamed, Alaa El-Din H. Sayed

**Affiliations:** 1 Department of Molecular Biology, Molecular Biology Research and Studies Institute, Assiut University, Assiut, 71516 Egypt; 2 Department of Zoology, Faculty of Science, Al-Azhar University, Assiut Branch, Assiut, 71524 Egypt; 3 Zoology Department, Faculty of Science, Assiut University, Assiut, 71516 Egypt

**Keywords:** Biochemistry, Physiology, Zoology

## Abstract

Microplastics are widely distributed in aquatic ecosystems along with other chemical pollutants. Therefore, it is vital to study the health-hazardous effects of MPs in combination with 4-nonylphenol (4-NP), which is a highly abundant industrial waste and a critical alkylphenol endocrine disruptor. We investigated the effects of the exposure to polyethylene microplastics (PE-MPs), 4-NP, and their combination on blood biomarkers in *Cyprinus carpio* juveniles. Four study groups were treated for 15 consecutive days: (1) control group, (2) 10 mg/L PE-MP group, (3) 10 mg/L PE-MPs + 200 µg/L 4-NP group, and (4) 200 µg/L 4-NP group, followed by 15 days of recovery. Biochemical analyses showed that creatine kinase, lactate dehydrogenase, glucose, liver enzymes, total protein, and A/G ratios were significantly increased after exposure to PE-MPs, 4-NP, and the combination. Hematological parameters (RBC's, Hb, Ht, neutrophil percentage, and WBC's) were significantly decreased in the three exposure groups, whereas mean corpuscular volume and lymphocyte percentages were significantly increased. The 15-day recovery period improved most hematobiochemical parameters and PE-MP accumulation indices. Taken together, we demonstrated the hazardous effects of PE-MP and 4-NP combinations on *C. carpio* blood parameters and highlighted their potential risk to human health.

## Introduction

Greater understanding of global pollution is warranted, especially in aquatic environments, and for animals, fish, and amphibians, where some industrial chemicals contaminate environments and cause serious damage during developmental and adult stages^1^. Chemical pollution is due to increased industrialization. Microplastics (MPs) and nonylphenols (NPs) are considered emerging pollutants and have attracted considerable environmental and research attention, but their combined toxicity toward aquatic organisms remains poorly researched. These chemicals are toxic when they persist in the environment and accumulate in different biota. Aside from their use in packaging, construction, transportation, electrical power, and medical products. Plastics are low cost, lightweight, and easy to process, which has made them popular in many industries^2^. Major MPs are categorized based on their monomer backbone structure and include polyethylenes (PEs), polypropylenes, polystyrenes, polyvinyl chlorides, and polyamides^3,4^. One of the most commonly used plastics is PE, which has the chemical formula (C_2_H_4_) n^5,6^. Many products are made from PE, including films, storage containers, toys, and packaging^7^. Plastics degrade during their life cycle via different mechanisms, including abrasion, mechanical wear, photooxidation, and biological destruction^8^. MPs are plastic pieces that vary across the size range from 5 to100 nm and are degraded from larger plastic pieces^9^, and NPs are < 100 nm in size^10^.

Despite differences in size, chemical content, and shape, MPs are heterogeneous groups of particles with varying toxicity^11,12^. Studies have shown that aquatic organisms ingest and accumulate MPs, concomitant contaminants^13,14^, and land invertebrates^15–17^. Although MPs come in many forms, the toxicity of each type is widely unknown^18–20^.

Certain species can be used to characterize the potential damage from MPs on aquatic health. Plastics may affect organisms in two ways: (i) physically by obstructing growth processes^21^ and reducing food and energy uptake^22,23^ and (ii) chemically by adsorbing contaminants such as polychlorinated biphenyls and polybrominated diphenyl ethers or releasing additives^9,24,25^. Freshwater is often the main source of such plastics, transporting medium, and the sink of MPs^26^. Little research has been conducted on MPs in freshwater when compared with marine water, but freshwater may accumulate numerous MPs^27^.

MPs also absorb other contaminants (organic and inorganic pollutants) from surrounding environments which is like the fact that these adhered pollutants are affected both spatially and biologically^24,28^ and warrants further study. Recently, this vector effect was summarized by Syberg et al.^29^, on base of the environment^30^, the organism^22^, and the cell (von Moos et al., 2012).

Persistent organic pollutants (POPs) are highly attracted to the hydrophobic surface of plastics, which facilitates the concentration of MPs^21^. However, it is highly unlikely that biota can absorb such chemicals from plastics^31^. Even though some POPs bioaccumulate and biomagnify within MPs, the risk of MPs-ingesting is generally the same as ingesting contaminants from prey or dispersed water contaminants^32–34^. The environmental endocrine disruptor nonylphenol (4-NP) is used in the manufacture of cleaning products, emulsifiers, and wetting agents and is found in paints, pesticides, and household products^35^.

Common carps (*Cyprinus carpio*) are a widely cultivated freshwater fish species because of their ability to withstand environmental changes and stresses. The species is widely used as a model species in ecotoxicological studies, so it was selected because of its importance in aquaculture^36,37^ using fish as biomarkers is vital for monitoring pollution. The hemato-biochemical parameters of fish are essential signs of water quality^38^.

Whereas, no previous studies have explored the synergistic effects of PE-MPs and 4-NP, especially in fauna from aquatic environments, we investigated the hematological and biochemical parameters in juvenile carp to determine the synergistic effects of PE-MPs and 4-NP using cytotoxicity biomarkers.

## Materials and methods

### Chemicals

#### PE-MP characterization

PE-MPs 5 mm > MPs > 100 nm were used in raw powdered form and were irregularly shaped particles (Toxemerge Pty Ltd., Australia). PE-MP particles were characterized using TEM (JEOL JEM-1200 EX II) at TEMU, Assiut University. PE-MPs were characterized using a protocol by Hamed et al.^39^.

#### 4-NP

4-NP (99.3% purity) was supplied by Sigma–Aldrich (Schnelldorf, Germany).

### Stock preparation

Approximately 1 g of PE-MPs and 6 mg of 4-NPs were individually dissolved into separate containers containing 1 L of distilled water and maintained in dark at 4 °C (stocks were shaken before use).

### PE-MP detection

PE-MPs were observed in whole fish based on a method by Deng et al.^40^. A whole fish (5 ± 1 g) was put in 10 mL of hydrogen peroxide (30%, v: v) for 2 h at 70 °C, and 100 µL of the obtained mixture was microscopely-examined using 14 MP OMAX camera (A35140U3) according to Hamed et al.^80^.

### Fish acclimation and exposure

Juveniles *C. carpio* (5 ± 1 g and 8.5 ± 1 cm) were acclimated in glass tanks (100 cm × 70 cm × 50 cm) under physicochemical conditions: Temperature 28.5 °C, pH 7.4, 6.9 mg/L DO, 12:12 h (light:dark), and 260.8 mM/cm conductivity. Four groups (36 fish/ 3 triplicate) were treated as follows: 1) control, 2) 10 mg/L PE-MPs, 3) 10 mg/L PE-MPs + 200 µg/L 4-NP, and 4) 200 µg/L 4-NP for 15 consecutive days and then 15 days of recovery. Doses were selected according to Hamed et al.^39^ and Sayed and Soliman^41^. Fish were fed each day with commercial pellets at 3% of their body weight, and water changed every day (50%) and MPs-redosed in water (immersion method of exposure) every day to prevent waste accumulation. Study procedures terminated with six fish in each group numbed on ice to eliminate stress caused by processing^42^ and fish was euthanized MS-222 (Millipore-Sigma-Aldrich, Oakville, ON, Canada; 0.5 g/L). After cutting the tail, blood collected in heparinized and non-heparinized tubes for hematological and biochemical assessments, respectively.

### Hematological parameters

Blood samples (6/ group) were taken from the caudal vein into heparinzed tubes to measure Hematological parameters including counts comprising erythrocytes (RBC's), total white blood cells (WBC's), differential WBC's, hematocrit (Ht), hemoglobin concentrations (Hb) were performed using Auto Hematology Analyzer (Rayto RT-7600) according to Hamed et al.^39^ and Hamed et al.^43^. Mean corpuscular volume (MCV), mean corpuscular hemoglobin (MCH), and mean corpuscular hemoglobin concentration (MCHC) were calculated using the formulae mentioned by Dacie and Lewis^101^

$${\text{MCHC }}\left( {{\text{g}}/{\text{dl}}} \right) \, = {\text{ Hb}}/{\text{ Ht }} \times { 1}00$$

$${\text{MCH }}\left( {{\text{pg}}} \right) \, = {\text{ Hb}}/{\text{ RBC}}^{\prime}{\text{s }} \times { 1}0$$

$${\text{MCV }}\left( {\mu {\text{m}}^{{3}} } \right) \, = {\text{ Ht}}/{\text{ RBC}}^{\prime}{\text{s }} \times { 1}0$$

### Biochemical parameters

Blood samples were taken from the caudal vein into non-heparinzed tubes to centrifugation at 5000 rpm for 5 min and then the serum was removed by subjecting the tubes, stored at − 20 °C until further analysis of the following blood parameters: albumin, globulin, total protein (TP), glucose, aspartate aminotransferase (AST), alanine aminotransferase (ALT), alkaline phosphatase (ALP), creatine kinase (CK), and lactate dehydrogenase (LDH) were determined by kits of (SGMitalia Company U.S.A) using a spectrophotometer (T80 + UV/VIS, Bioanalytic Diagnostic Industry, Co.) according to Hamed et al.^43^.

### Erythrocyte analysis

Blood smears fixed, air-dried, and stained with hematoxylin & eosin. Slides were selected and observed under a 40 × objective lens under a light microscope (VE-T2) attached to a 14 MP OMAX camera (A35140U3) according to Hamed et al.^43^ and Sayed et al.^44^**.**

### Statistical analysis

The mean and standard error of the mean values were estimated. Statistical differences between groups were analyzed by one-way analysis of variance in SPSS^45^ at the 0.05 significance level (*P* < 0.05). Post hoc comparison was done using Tukey’s-b and Dunnett tests (SPSS V.16).

### Ethics statement

Studies were approved by the Research Ethics Committee of the Molecular Biology Research and Studies Institute (MB-21–11-R), Assiut University, Assiut, Egypt. All methods were carried out following the relevant regulations and ARRIVE guidelines.

## Results

### PE-MP characterization

**Figure 1:**
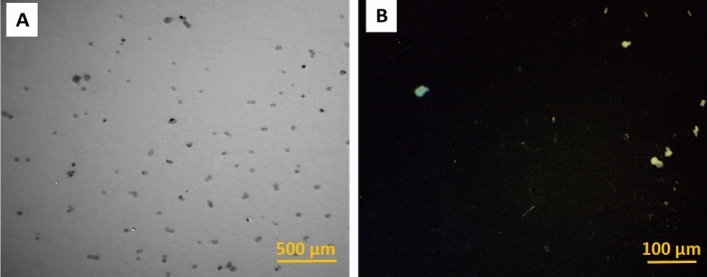
(**a**) Transmission electron microscope and (**b**) light microscope images of PE-MPs.

Light microscope and TEM images showed that PE-MP particles were irregularly shaped (Fig. 1).

### PE-MP detection

**Figure 2:**
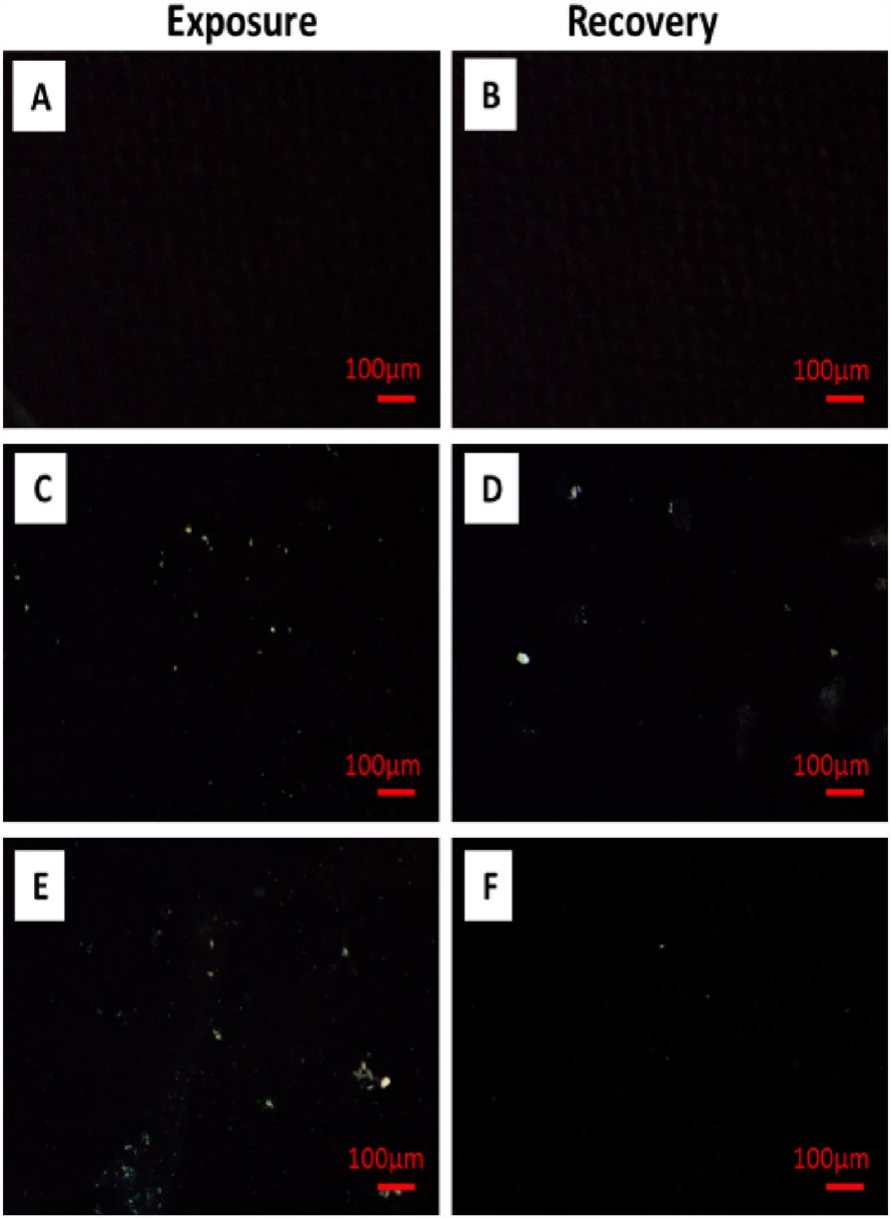
Light microscope images showing PE-MPs in fish, (**a**, **b**) Control, (**c**, **d**) 10 mg/L PE-MPs, and (**e**, **f**) 10 mg/L PE-MPs + 200 µg/L 4-NP.

High PE-MP levels were observed in the PE-MP-exposed groups when compared with the control. After the recovery period, high PE-MP levels were still observed in the PE-MP-exposed groups compared with control (Fig. 2).

### The effects of combinations on hematological parameters

**Figure 3:**
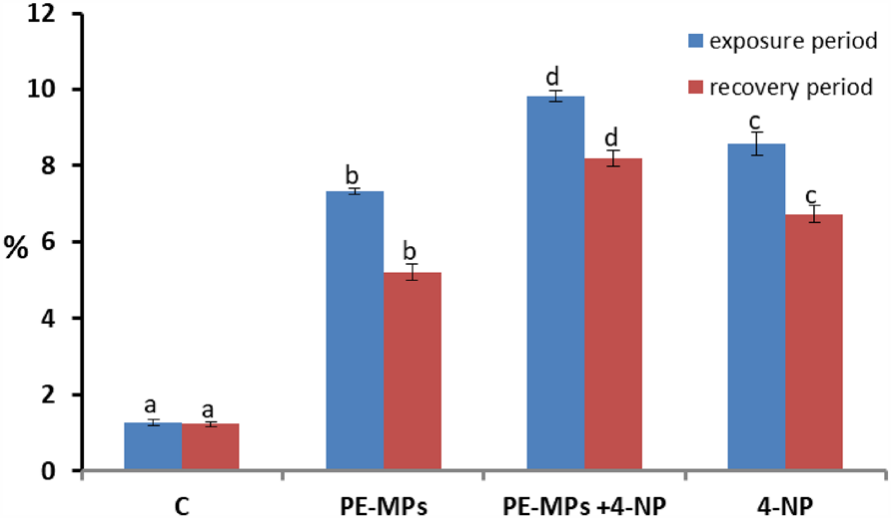
RBC's percentage alterations in juveniles *Cyprinus carpio* after exposure to 10 mg/L PE-MPs, 10 mg/L PE-MPs + 200 µg/L 4-NP, and 200 µg/L 4-NP for 15 days each and after 15 days of recovery. Different letters indicate significant differences (*P* < 0.05).

**Figure 4:**
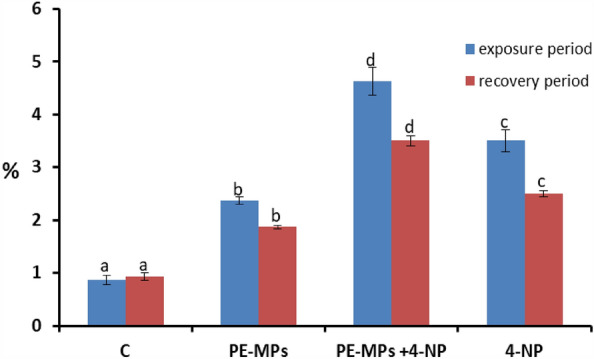
RBC's nuclear abnormality percentages in juveniles *Cyprinus carpio* after exposure to 10 mg/L PE-MPs, 10 mg/L PE-MPs + 200 µg/L 4-NP, and 200 µg/L 4-NP for 15 days each and after 15 days of recovery. Different letters indicate significant differences (*P* < 0.05).

RBC's, Hb, and WBC's after exposure to 10 mg/L PE-MPs, 200 µg/L 4-NP, and 10 mg/L PE-MPs + 200 µg/L 4-NP; Ht after exposure to 200 µg/L 4-NP and 10 mg/L PE-MPs + 200 µg/L 4-NP; and neutrophil percentages after exposure to 200 µg/L 4-NP for 15 days showed significant (*P* < 0.05) decreases when compared with those of controls. MCV levels and lymphocyte percentages showed significant (*P* < 0.05) increases after exposure to 10 mg/L PE-MPs + 200 µg/L 4-NP for 15 days (Figs. 3 and 4).

Neutrophil and monocyte percentages and Ht levels after exposure to 10 mg/L PE-MPs, and monocyte and lymphocyte percentages and MCH levels after exposure to 10 mg/L PE-MPs + 200 µg/L 4-NP for 15 days showed nonsignificant decreases. MCHC levels after exposure to 10 mg/L PE-MPs, 200 µg/L 4-NP, and 10 mg/L PE-MPs + 200 µg/L 4-NP; neutrophil percentages and MCV levels after exposure to 10 mg/L PE-MPs + 200 µg/L 4-NP; monocyte percentages after exposure to 200 µg/L 4-NP; and MCH levels after exposure to 200 µg/L 4-NP and 10 mg/L PE-MPs for 15 days showed nonsignificant increases.

After the recovery period, the hematological parameters showed no change, except for the following: the RBC, MCV, and MCH levels in the PE-MP group, MCHC levels and neutrophil percentages in the PE-MPs + 4-NP group, and monocyte percentages in the 4-NP group; all showed nonsignificant decreases. The MCV levels in the 4-NP group showed a nonsignificant increase. The Ht levels in the PE-MP group showed a significant decrease. The lymphocyte percentages in the PE-MP + 4-NP group showed a significant increase (Table 1).

**Table 1:** The effects of PE-MPs, 4-NP, and PE-MPs + 4-NP on hematological parameters (mean ± standard error) of juveniles *Cyprinus carpio* after 15 days of exposure, followed by 15 days of recovery.

	Exposure period				Recovery period			
	Control	PE-MPs	PE-MPs + 4-NP	4-NP	Control	PE-MPs	PE-MPs + 4-NP	4-NP
RBCs (million/µL)	1.46 ± 0.02^c^	1.37 ± 0.01^b^	1.29 ± 0.01^a^	1.34 ± 0.01^b^	1.47 ± 0.02^b^	1.43 ± 0.02^b^	1.32 ± 0.02^a^	1.33 ± 0.02^a^
Hemoglobin (Hb)(g/dL)	9.58 ± 0.08^c^	9.07 ± 0.06^b^	8.34 ± 0.17^a^	9.13 ± 0.01^b^	9.63 ± 0.08^c^	9.15 ± 0.10^b^	8.33 ± 0.21^a^	9.10 ± 0.00^b^
Ht (PCV) (%)	39.24 ± 0.24^c^	38.97 ± 0.30^bc^	35.57 ± 0.44^a^	37.88 ± 0.29^b^	39.38 ± 0.41^c^	37.13 ± 0.48^b^	35.55 ± 0.53^a^	37.52 ± 0.18^b^
MCV (µm^3^)	268 ± 3.79^a^	284. ± 2.66^b^	276 ± 3.95^ab^	284 ± 3.06^b^	270 ± 4.27^a^	262 ± 3.54^a^	271 ± 5.96^a^	277 ± 3.74^a^
MCH (µm^3^)	65.47 ± 0.61^a^	66.18 ± 0.48^a^	64.80 ± 1.91^a^	68.37 ± 0.35^a^	65.90 ± 1.14^a^	64.53 ± 0.93^a^	63.48 ± 2.07^a^	67.25 ± 0.82^a^
MCHC (%)	20.75 ± 3.60^a^	23.27 ± 0.15^a^	23.47 ± 0.63^a^	24.10 ± 0.20^a^	24.47 ± 0.32^a^	24.68 ± 0.37^a^	23.45 ± 0.79^a^	24.30 ± 0.13^a^
WBCs (thousand/µL)	53.67 ± 0.33^c^	51.67 ± 0.33^b^	49.83 ± 0.54^a^	52.09 ± 0.42^b^	54.33 ± 0.42^c^	51.50 ± 0.34^ab^	50.50 ± 0.43^a^	52.17 ± 0.17^b^
Neutrophils (%)	15.33 ± 0.21^bc^	14.50 ± 0.22^ab^	16 ± 0.26^c^	13.83 ± 0.31^a^	15.67 ± 0.21^c^	14.33 ± 0.21^ab^	15 ± 0.26^bc^	13.83 ± 0.17^a^
Lymphocytes (%)	83 ± 0.26^a^	84 ± 0.00^b^	82.67 ± 0.42^a^	84.33 ± 0.21^b^	82.83 ± 0.17^a^	84.17 ± 0.31^b^	83.83 ± 0.40^b^	84.83 ± 0.17^b^
Monocytes (%)	1.67 ± 0.21^a^	1.50 ± 0.22^a^	1.33 ± 0.21^a^	1.83 ± 0.17^a^	1.67 ± 0.21^a^	1.50 ± 0.22^a^	1.17 ± 0.17^a^	1.17 ± 0.17^a^

Different superscript letters indicate significantly different (*P* < 0.05).

### The effects of combinations on biochemical parameters

AST, ALT, ALP, LDH, CK, glucose, and TP after exposure to 10 mg/L PE-MPs, 200 µg/L 4-NP, and 10 mg/L PE-MPs + 200 µg/L 4-NP, and globulin and albumin after exposure to 10 mg/L PE-MPs and 10 mg/L PE-MPs + 200 µg/L 4-NP showed significant (*P* < 0.05) increases. By contrast, albumin and the A/G ratio after exposure to 200 µg/L 4-NP showed a nonsignificant increase. The A/G ratio after exposure to 10 mg/L PE-MPs + 200 µg/L 4-NP showed a nonsignificant decrease.

After the recovery period, the biochemical parameters did not change, except for the following: TP and AST in the PE-MPs, 4-NP, and PE-MPs + 4-NP groups; LDH and glucose in the 4-NP-exposed group; and albumin in the PE-MP-exposed group showed nonsignificant increases.

Additionally, the A/G ratio in the 4-NP group and globulin in the PE-MP group showed a nonsignificant decrease. However, the A/G ratio in the PE-MPs + 4-NP group and albumin in the 4-NP group showed a significant decrease (*P* < 0.05) (Table 2).

**Table 2:** The effects of PE-MPs, 4-NP, and PE-MPs + 4-NP on biochemical parameters (mean ± standard error) of juveniles *Cyprinus carpio* after 15 days of exposure, followed by15 days of recovery.

	Exposure period				Recovery period			
	Control	PE-MPs	PE-MPs + 4-NP	4-NP	Control	PE-MPs	PE-MPs + 4-NP	4-NP
LDH (U/L)	74.08 ± 0.66^a^	79.80 ± 1.59^b^	87.15 ± 0.76^c^	78.62 ± 0.42^b^	74.12 ± 0.56^a^	84.68 ± 2.52^b^	86.12 ± 0.69^b^	77.62 ± 0.69^a^
Creatine kinase (CK) (U/L)	82.85 ± 0.42^a^	86.37 ± 0.46^b^	86.75 ± 0.30^b^	85.28 ± 0.65^b^	81.80 ± 0.19^a^	85.72 ± 0.45^b^	85.82 ± 0.50^b^	84.28 ± 0.55^b^
ALP (U/L)	8.19 ± 0.02^a^	8.59 ± 0.04^b^	8.87 ± 0.05^c^	8.53 ± 0.05^b^	8.21 ± 0.05^a^	8.67 ± 0.04^b^	8.83 ± 0.04^c^	8.53 ± 0.03^b^
ALT (U/L)	48.42 ± 0.48^a^	51.23 ± 0.31^b^	53.05 ± 0.70^b^	51.40 ± 0.35^b^	48.35 ± 0.30^a^	51.28 ± 0.25^b^	52.90 ± 0.34^c^	51.42 ± 0.14^b^
AST (U/L)	82.83 ± 0.41^a^	85.25 ± 0.30^b^	86.30 ± 0.38^b^	85.75 ± 0.50^b^	84.30 ± 0.54^a^	85.62 ± 0.44^a^	85.30 ± 0.48^a^	85.65 ± 0.31^a^
Glucose (mg/dL)	55.73 ± 0.38^a^	58.12 ± 0.35^b^	58.68 ± 0.19^b^	57.40 ± 0.49^b^	55.27 ± 0.30^a^	57.33 ± 0.64^bc^	58.20 ± 0.36^c^	55.85 ± 0.34^ab^
Total protein (g/dL)	2.73 ± 0.04^a^	2.93 ± 0.02^b^	2.94 ± 0.02^b^	2.88 ± 0.02^b^	2.78 ± 0.03^a^	2.87 ± 0.02^a^	4.03 ± 1.17^a^	2.83 ± 0.02^a^
A/G ratio (%)	0.34 ± 0.01^a^	0.34 ± 0.00^a^	0.33 ± 0.00^a^	0.35 ± 0.00^a^	0.35 ± 0.00^a^	0.35 ± 0.00^a^	0.27 ± 0.04^a^	0.32 ± 0.02^a^
Globulin (g/dL)	2.15 ± 0.01^a^	2.24 ± 0.03^b^	2.41 ± 0.02^c^	2.15 ± 0.02^a^	2.13 ± 0.01^a^	2.10 ± 0.03^a^	3.35 ± 0.63^a^	2.13 ± 0.03^a^
Albumin (g/dL)	0.74 ± 0.01^a^	0.77 ± 0.01^a^	0.81 ± 0.01^b^	0.76 ± 0.01^a^	0.76 ± 0.01^b^	0.77 ± 0.02^b^	0.80 ± 0.00^b^	0.70 ± 0.00^a^

Different superscript letters indicate significantly different (*P* < 0.05).

### Erythrocyte morphological alterations and nuclear abnormalities

**Figure 5:**
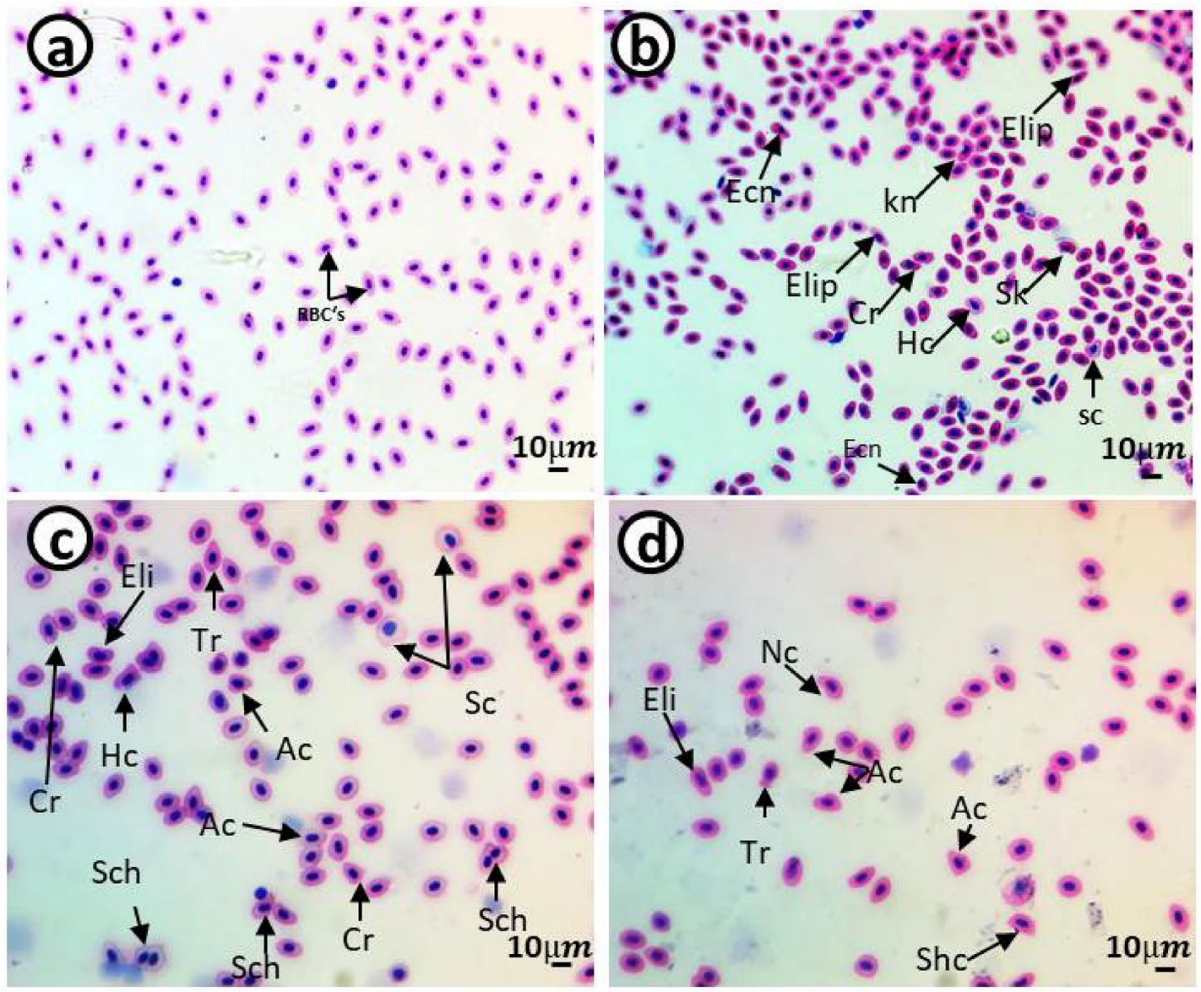
Blood smears from juveniles *Cyprinus carpio* after a 15-day exposure showing normal erythrocytes in the control group (**a**) and deformed erythrocytes in the 10 mg/L PE-MPs (**b**), 10 mg/L PE-MPs + 200 µg/L 4-NP (**c**), and 200 µg/L 4-NP groups (**d**). Eliboat shapes (Eli), teardrops (Tr), schistocytics (Sch), swollen cells (Sc), eccentric nucleus (Ecn), kidney shapes (Kn), crenated cells (Cr), sickle cells (Sk), acanthocytes (Ac), vacuolated cells (Vc), elliptocytes (Elip), spinocytes (Spc), and hemolyzed cells (Hc) (hematoxylin & eosin staining).

**Figure 6:**
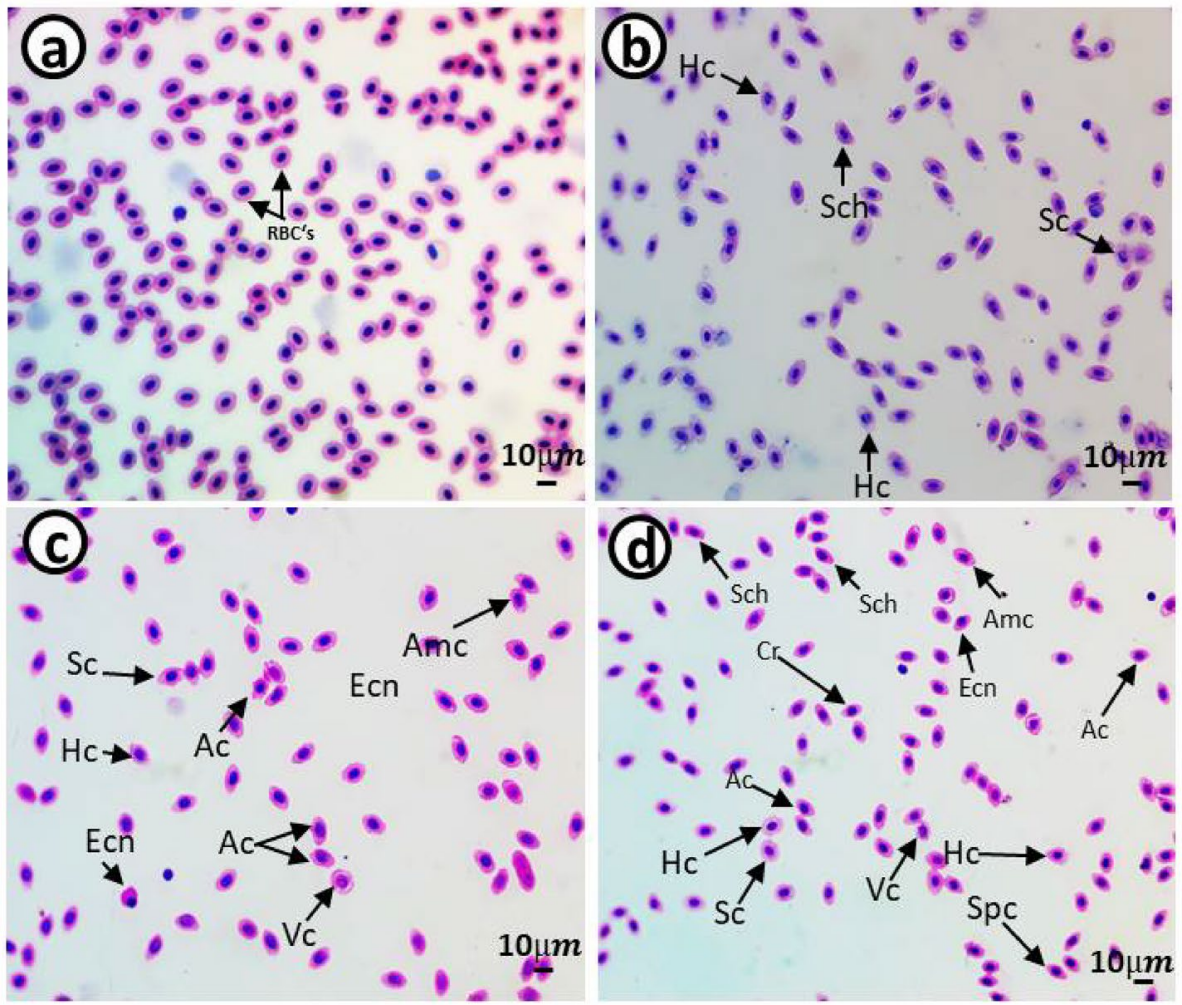
Blood smears from juveniles *Cyprinus carpio* after a recovery period of 15 days showing normal erythrocytes in the control group (**a**) and deformed erythrocytes in the 10 mg/L PE-MPs (**b**), 10 mg/L PE-MPs + 200 µg/L 4-NP (**c**), and 200 µg/L 4-NP groups (**d**). Schistocytic (Sch), swollen cells (Sc), eccentric nucleus (Ecn), crenated cells (Cr), acanthocytes (Ac), vacuolated cells (Vc), ameboied cells (Amc), spinocytes (Spc), and hemolyzed cells (Hc) (hematoxylin & eosin staining).

The control group showed a standard RBC's shape with a centrally located nucleus (Figs. 5a and 6a). Smears from the 10 mg/L PE-MP (Figs. 5b and 6b), 10 mg/L PE-MPs + 200 µg/L 4-NP (Figs. 5c and 6c), and 200 µg/L 4-NP groups displayed poikilocytosis in RBC's (Figs. 5d and 6d) after exposure, whereas after recovery, cells showed varied morphologies, including eliboat shapes, teardrops, schistocytic, swollen cells, eccentric nuclei, kidney shapes, eliptocytes, crenated shapes, sickle cells, acanthocytes, hemolyzed cells, vacuolated cells, ameboied cells, and spinocytes. The percentages of RBC alterations and nuclear abnormalities were significantly increased (*P* < 0.00001) after exposure to 10 mg/L PE-MPs, 200 µg/L 4-NP, and 10 mg/L PE-MPs + 200 µg/L 4-NP for 15 days when compared with controls. RBC's membrane and nuclei alterations remained significantly increased after the 15-day recovery period when compared with controls (Figs. 3 and 4). After both periods, percentage increments (for both types of alterations) followed the order: PE-MPs + 4-NP > 4-NP > PE-MPs.

## Discussion

MP contamination in aquatic environments is a growing hazardous health problem as aquatic animals eat and digest MPs. Living organisms may be affected by MPs increasing their bioavailability and uptake of sorbed co-contaminates of different types. The combined effects of MPs and sorbed co-contaminants in aquatic organisms remain to be fully verified^46^. Therefore, in this study, hemato-biochemical parameters were used to investigate the effects of *C. carpio* exposure to 10 mg/L PE-MPs, 200 µg/L 4-NP, and 10 mg/L PE-MPs + 200 µg/L 4-NP for 15 days and then 15 days of recovery.

To assess fish health, hematological parameters must be measured^47^. We observed considerable variations in hematological parameters between groups. Our results were similar to the data reported by Hamed et al.^39^ and Hamed et al.^43^. Decreased RBC, Hb, Ht, and WBC levels with increased MCV levels and lymphocyte percentages were also reported by Hamed et al.^39^ in *Oreochromis niloticus* exposed to MPs and by^43^ in *C. carpio* exposed to PE-MPs. Additionally, in fish exposed to phosalone^48^, and copper oxide nanoparticles^49^. The catfish (*Clarias gariepinus*) was also affected by UVA exposure with respect to hematology and cell alterations^50^. Some of parameters with hydroxychloroquine in catfish (*C. gariepinus*), Sayed et al.^44^ and Mekkawy et al.^51^, and Abou Khalil et al.^52^ with African catfish *(C. gariepinus) *in the presence of 4-NP. The exposure of carp to PE-MPs, 4-NP, and PE-MPs + 4-NP caused anemia, which might have been attributed to hematopoietic tissue alterations^53^ and^54^. This possibly occurred because of increased mechanical fragility in cell membranes, which we observed in our erythrocyte morphology^55^. Under laboratory conditions and after exposure to different pollutants, peripheral RBCs, Hb, and Ht were decreased^56^, which might have been due to the heme-dilution of blood which resulted from tissue damage^57^. The negative effects of NPs on lymphoid tissues in exposed fish may reduce total WBC counts^58^. The bioaccumulation of pollutants in tissues may decrease WBCs. As blood oxygen levels decrease, toxicity caused by plastics could be enhanced by decreased hemoglobin levels. Similar results were reported by Mukherjee and Sinha^59^ as cadmium contamination response^39^ as effect of MPs and^43^ as an effect of PE-MPs. Damage to the immune system after MPs accordingly will cause damage to the defense system and animal health^60^.

Biochemical parameters are invaluable bioindicators^41,56^. Most biochemical parameters were significantly increased after exposure to PE-MPs, 4-NP, and PE-MPs + 4-NP when compared with controls and were consistent with Hamed et al.^43^. The rise in CK, LDH, ALP, ALT, AST, TP, glucose with variation by increase and decrease in albumin and globulin observed in our study were similarly studied by Hamed et al.^39^ with *O. niloticus* in the presence of MPs. Some parameters with hydroxychloroquine in catfish *(C. gariepinus)*^44^, ^61^ and^41^ with African catfish in the presence of 4-NP, and^43^ with common carp (*C. carpio*) in the presence of PE-MPs. Banaee et al.^62^ and Nematdoost Haghi and Banaee^63^ state that the higher levels of the enzymes (CK, AST, ALT, LDH, and ALP) in serum are regarded as biomarker for cell membranes damage and as a tool for diagnosing changes in the environment in ecotoxicological studies, these enzymes are indicative of lesions in the tissues. Furthermore, elevated glucose levels indicated that glycogen had disintegrated or that its absorption was restricted in the liver. By contrast, increased blood glucose levels may have been due to hepatic tissue glycogen disintegration or impaired glucose absorption^64^. Previously, damage to different organ membranes in fish was observed following increased enzyme activity (ALT, AST, and ALP) caused by paraquat and plastic particles^65^, and in *Pomatoschistus microps*, biochemical parameters were changed when exposed to MPs and pyrene or to MPs and/or nickel^66^. Body homeostasis is maintained by proteins that prevent fluid leakage throughout the body^60^.

Immune system diseases and other kidney and hepatic issues are assessed using TP, albumin, and globulin tests^67^. Stoyanova et al.^68^ reported that the intensification of anaerobic metabolism could be measured by LDH activity due to environmental changes, pollution, and energy depletion. Changes in LDH, AST, ALT, CK, and ALP activities were shown to indicate tissue lesions and to reflect environmental changes in ecotoxicology^69^. ALT and AST levels were increased following hepatocyte damage (Komatsu et al., 2002) or impaired carbohydrate and protein metabolism^70^. Peralta et al.^71^ and Ramos-Barron et al.^72^ reported that increased albumin levels indicated hepatorenal tissue alterations. Wiegertjes et al.^73^ demonstrated that increased globulin levels were viable immune responses. In Nile tilapia (*O. niloticus*) exposed to MPs, albumin, globulin, and TP levels were higher, potentially indicating a damaged liver^39^ and in *C. carpio* induced by PE-MPs^43^. Osman et al.^50^ showed that fish underwent hyperglycemia when exposed to UVA stress or heavy metals and other contaminants^74^.

High PE-MPs levels were observed in PE-MP-exposed fish when compared with controls. This may have been due to the entrance of plastic particles with the water flow to the fish's body. A significant concentration of MPs was reported in different zebrafish organs^75^. Additionally, significantly higher MPs were observed in *O. niloticus* after MP exposure for 15 days^39^. MP accumulation in zebrafish yolk sac and migration to other organs were observed during embryogenesis^76^. Furthermore, in mussels, MPs absorbed through the gut mucosa were transported through the bloodstream to different tissues^77^. Moreover, PS-MP bioaccumulation was dose- and time-dependent in *O. niloticus*^78^.

The percentage of erythrocytes with morphological alterations and nuclear abnormalities, when exposed to treatments, was significantly increased when compared with controls. Poikilocytosis may be affected by several factors, e.g., increased RBC membrane fluidity, declined ATP levels, and inhibited membrane-bound enzymes^79^. Our results were similar to other fish pollutant studies: Hamed et al.^43^, who studied *C. carpio* exposed to PE-MPs; Sayed et al.^44^, who studied *C*. *gariepinus* exposed to hydroxychloroquine; Hamed et al.^80^, who studied the protective role of *Spirulina platensis* against cytotoxicity and genotoxicity induced by lead nitrates in *C. gariepinus*; Soliman et al.^49^, who studied the damage caused by copper sulfate and copper oxide nanoparticles in *O. niloticus*; and Sayed et al.^81^, who investigated *Oryzias latipes* exposed to 4-NP. Several studies reported that morphological and nuclear abnormalities in erythrocytes and MN were genotoxicity biomarkers following exposure to radiation and chemicals^51,82–89^. These biomarkers are powerful assessment tools for genetic and cellular damage in eukaryotes as they reflect DNA damage, and are simple, reliable, and sensitive measurement tools^51,84,86,87,90^.

Our blood parameter data indicated that the damage caused by combined PE-MPs and 4-NP was higher than the damage caused by 4-NP alone, which may be due to MPs facilitating entrance of other contaminants into aquatic organisms^24,29,91^. Previous laboratory studies reported that MPs were ingested by aquatic organisms^77,92^. Besseling et al.^22^, Koelmans et al.^93^, and Chua et al.^94^ reported that MPs may carry other contaminants, including plasticizers and POPs, such as polychlorinated biphenyls and polycyclic aromatic hydrocarbons^65,93,95,96^, and could facilitate interactions with metals, such as PE-MP-mediated silver uptake in *Danio rerio*^97^. Some ecotoxicological studies have provided evidence of metal ion adsorption by plastic containers^98,99^. HOCs strongly and chemically sorb onto MPs than natural sediments according to a study comparing sorption rates onto natural and manufactured particulates^30^. Oliveira et al.^65^ reported that PE bead exposure significantly increased toxicant bioavailability in juveniles exposed to lethal pyrene concentrations.

After the 15-day recovery period, erythrocyte morphological alterations, nuclear abnormalities, PE-MPs, and hematobiochemical changes were apparent in all treated groups when compared with controls. Our results were supported by Martins and Guilhermino^100^, who reported that despite the depuration phase, MPs persisted in *D. magna* for many generations, whereas Hamed et al.^39^ indicated that MPs were detected and generated hematobiochemical effects in Nile tilapia after the recovery period. Notably, after recovery, a faint improvement in some parameters was observed but was not similar to controls. A possible reason for this could be that a 15-day recovery was not enough for fish to eliminate the toxic effects of pollutants or the tissue has bo ability to restore their functions as normal after MPs-exposure^46^.

## Conclusions

The synergistic effect of PE-MPs and 4-NP induced a high degree of increase in creatine kinase, lactate dehydrogenase, glucose, liver enzymes, total protein, and A/G ratios after exposure to PE-MPs, 4-NP, and the combination. Also, hematological parameters (RBC's, Hb, Ht, neutrophil percentage, and WBC's) were significantly decreased in the three exposure groups. The 15-day recovery period improved most hematobiochemical parameters and PE-MP accumulation indices. Hematological and biochemical issues in carp when compared with individual exposures, our data showed that the synergistic effect of PE-MP and 4-NP caused more serious damage than each single chemical in dose dependent manner.

## Data Availability

All data generated or analyzed during this study are included in the research article.
